# Customized Novel Design of 3D Printed Pregabalin Tablets for Intra-Gastric Floating and Controlled Release Using Fused Deposition Modeling

**DOI:** 10.3390/pharmaceutics11110564

**Published:** 2019-10-30

**Authors:** Shrawani Lamichhane, Jun-Bom Park, Dong Hwan Sohn, Sangkil Lee

**Affiliations:** 1 College of Pharmacy, Keimyung University, 1095 Dalgubeol-daero, Dalseo-gu, Daegu 42601, Korea; phr.shrawani@gmail.com (S.L.); dhsohn@kmu.ac.kr (D.H.S.); 2 College of Pharmacy, Samyook University, 815 Hwarang-ro, Nowon-gu, Seoul 01795, Korea; junji4@gmail.com

**Keywords:** 3D printing, FMD, pregabalin, controlled release, gastric floating

## Abstract

Three-dimensional (3D) printing has been recently employed in the design and formulation of various dosage forms with the aim of on-demand manufacturing and personalized medicine. In this study, we formulated a floating sustained release system using fused deposition modeling (FDM). Filaments were prepared using hypromellose acetate succinate (HPMCAS), polyethylene glycol (PEG 400) and pregabalin as the active ingredient. Cylindrical tablets with infill percentages of 25%, 50% and 75% were designed and printed with the FDM printer. An optimized formulation (F6) was designed with a closed bottom layer and a partially opened top layer. Filaments and tablets were characterized by means of fourier-transform infrared spectroscopy (FTIR), differential scanning calorimetry (DSC), X-ray powder diffraction (XRPD), and thermogravimetric analysis (TGA). The results show that the processing condition did not have a significant effect on the stability of the drug and the crystallinity of the drug remained even after printing. A dissolution study revealed that drug release is faster in an open system with low infill percentage compared to closed systems and open systems with a high infill ratio. The optimized formulation (F6) with partially opened top layer showed zero-order drug release. The results show that FDM printing is suitable for the formulation of floating dosage form with the desired drug release profile.

## 1. Introduction

The concept of three-dimensional (3D) printing has been flourishing since the 1980s and has been applied to various fields as a tool for rapid prototyping, custom manufacturing and complex manufacturing [1]. Contrasting conventional manufacturing technique, 3D printing involves the fabrication of a 3D structure layer-by-layer from the bottom using a digital design, hence, it is also known as additive manufacturing [2,3]. From products as simple as hearing aids to high-tech parts of military jets, the scope of 3D printing is growing rapidly [4,5,6]. Furthermore, the excellence of this technology is not new in the medical and pharmaceutical sectors. From educational tools to surgical instruments to printed organs for transplantation, 3D printing is evolving as a new tool in the medical sector [7]. Likewise, FDA approval of the first 3D printed medicine in 2016 for the treatment of epilepsy has led to confidence that this technology can cause a paradigm shift in the field of pharmaceutics [3]. Several studies have been conducted and proven to show the suitability of using printing technology to develop different dosage forms with variable drug release profiles [4]. In recent years, numerous 3D printing technologies have been introduced and have been exploited for their respective advantages and disadvantages. Stereolithography was the first technology to be developed as a rapid prototyping technique [1], followed by fused deposition modeling (FDM), selective laser sintering (SLS) [8,9,10,11], and binder jet printing. Binder jet printing or inkjet printing was one of the first 3D printing technologies to be used in the preparation of drug delivery devices and one of the most widely studied technologies to date [12]. Most of the technologies involve a high temperature, which is one of the major drawbacks of these processes, especially in the case of pharmaceuticals. Nevertheless, this technology has provided a new method for the preparation of personalized medicine with an accurate and adjustable dose and customized drug release profiles. Moreover, complex formulations such as combined dosage forms with mixed release kinetics and complex designs have been carried out to achieve better patient compliance and better therapeutic outcomes. FDM, a technology developed in the late 1980s, is also one of the most widely studied technologies in pharmaceutics. This technology, based on material extrusion, involves melting of filaments and deposition of the melted materials in layers where they fuse together to fabricate a 3D structure [4,13]. For this method, the materials used should be thermostable, non-volatile and non-aerosolizing [14]. Commercially used polymers include polylactic acid or polylactide (PLA), polyvinyl alcohol (PVA) and acrylonitrile butadiene styrene (ABS) [3,15]; however, in the case of pharmaceuticals, polymers like Eudragit^®^, hydroxypropyl cellulose (HPC), and hypromellose^®^ (HPMC) have been studied [16,17]. Initial studies also involved drug-loaded PVA filaments prepared by soaking commercial filaments in alcoholic drug solutions [18,19,20,21]. These studies proved the suitability of preparing various modified dosage forms using FDM technology, after which several pharmaceutical polymers along with suitable plasticizers were used to prepare filaments. Recently, a modification of fused deposition modeling has been introduced, known as direct powder extrusion, which works on a similar principle, minimizing the need to prepare filaments [22]. However, FDM technology is still being extensively studied in the field of drug delivery and dosage forms design [23,24]. One of the major advantages of this technology is its cost-effectiveness, along with its availability compared to other 3D printing technology. In contrast, FDM incorporates the deposition of materials vertically layer by layer, resulting in a step-like surface which appears relatively rougher compared to other technologies. Nevertheless, the resolution of printing does not affect the drug release mechanism; hence, it is suitable for the fabrication of desired dosage forms. 

Pregabalin, a chemical analogue of neurotransmitter gamma-aminobutyric acid (GABA), is an α_2_δ receptor agonist which has analgesic, anticonvulsant and anxiolytic activities [25,26]. Immediate release formulations of pregabalin, available in different dosage strength, are approved by the FDA for the management of diabetic peripheral neuropathy, fibromyalgia, post-herpetic neuralgia, and also as adjunctive therapy for partial seizures [27]. However, pregabalin has a short elimination half-life of approximately 6 h [28]. Due to this reason, a commercial immediate release form must be administered 2–3 times a day. In 2017, the US FDA approved a sustained release of once daily tablets for the management of diabetic peripheral neuropathy and fibromyalgia. Controlled release pregabalin was found to be effective in reducing pain with a similar safety profile to that of immediate release pregabalin [29]. According to the biopharmaceutics classification system (BCS), pregabalin is a class I compound with high solubility and high permeability. Moreover, the drug has been proven to be mainly absorbed in the stomach and upper gastrointestinal tract [30]. Thus, one of the ways to decrease the frequency of administration has been to increase the gastric retention of the formulation. Among the various approaches for increasing the gastric retention time of the dosage form, only floating and swelling mechanisms have shown clinical evidence for prolonged gastric residence time at fed state. Floating gastro-retentive formulations can effectively minimize the risk of premature gastric emptying of swellable systems by floating above gastric juice and being away from the pylorus [27]. Previously, an intragastric floating tablets of domperidone was formulated using 3D technology. As FDM 3D technology requires filaments as the main material, hydroxy-propyl cellulose filaments loaded with domperidone were prepared using hot melt extruder and hollow tablets were made using a 3D printer. Printed tablets were studied for in vitro and in vivo floating time and drug release profile, which demonstrated a promising application of FDM technology to reduce the frequency of administration and improve patient compliance [31]. Moreover, various pharmaceutical grade filaments have been prepared in previous studies [17]. In this study, by combining these studies, we aimed to design a floating tablet of pregabalin with a controlled drug delivery profile using 3D printing. To the best of our knowledge, this is the first study to employ FDM technology to formulate a floating controlled release system using a novel shape of a tablet and pregabalin as a model drug. 

## 2. Materials and Methods 

### 2.1. Materials

Pregabalin and hypromellose (HPMC E4) were kindly donated from GL PharrmTech, Republic of Korea. Hot melt extrudable (HME) grade hypromellose (HPMC HME 15LV) was purchased from Colorcon, Seoul, Korea. Polyethylene glycol (PEG 400) was purchased from Yakuri pure chemicals Co., Ltd. (Kyoto, Japan). And hypromellose acetate succinate (HPMCAS, AQUOT AS-HG) was purchased from Shin-Etsu chemical Co., Ltd., Tokyo, Japan. Ammonium phosphate dibasic, sodium 1-octanesulfonate and polyvinyl alcohol (PVA) were purchased from Sigma-aldrich, Seoul, Republic of Korea. HPLC grade methanol and acetonitrile were purchased from Duksan chemicals, Seoul, Korea. Commercially available Lyrica^®^ CR165 (Pfizer Inc., New York, NY, USA) was used as the reference product for the in vitro release study.

### 2.2. Preparation of Pregabalin-Loaded Filaments

Pregabalin-loaded filaments were prepared using Process 11 twin screw Hot Melt Extruder (Thermo scientific, Waltham, MA, USA) with nozzle diameter of 1.5 mm. Physical blends of active pharmaceutical ingredient (API) and polymers were prepared in a mixer and filaments were extruded at a temperature of 125 °C at a rotational speed of 10–20 rpm with a torque of 50–60 nm and used for the preparation of tablets.

### 2.3. Design and Printing of Tablets

**Figure 1:**
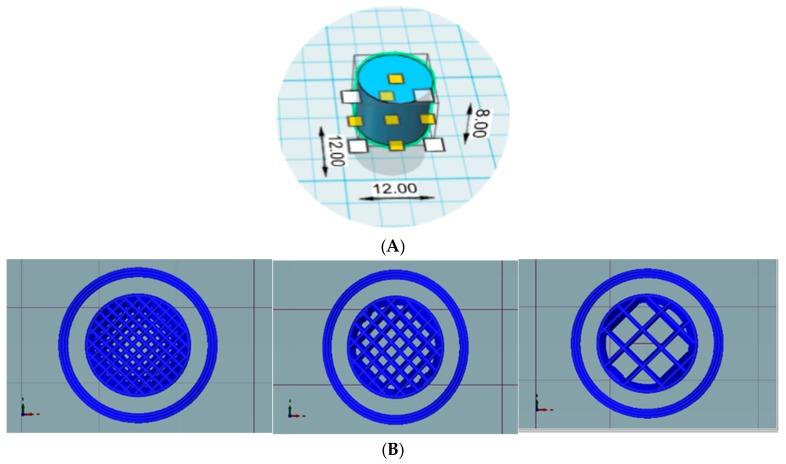
Design and internal structures of tablets. Design of preliminary cylindrical tablets (**A**) and slicing of tablets with infill percentages of 25%, 50% and 75%, left to right (**B**).

Tablets were designed using Autodesk^®^ 123D^®^ design software version 1.1.4. (Autodesk, San Rafael, CA, USA) Cylindrical tablets were designed for preliminary studies and a novel shape of tablets was designed for optimized formulation. Printed tablets were then sliced using a slicing software Repetier host version 2.1.3 (Hot-World GmbH & Co. Willich, Germany) with an in-built slicer Cura engine. Finally, the tablets were printed using a Good bot 4025-MP FDM printer (3D Korea, Yongsin-ri, Republic of Korea) with a brass nozzle with a diameter of 0.2 mm. The printing temperature was 180 °C and the bed temperature was 50 °C, which remained constant for all the formulations. Various tablets were printed with different infill percentages as open or closed system. Open system tablets did not have a top and bottom layer, whereas closed system tablets had top and bottom layers of a thickness of 0.4 mm (Figure 1). Shell thickness was kept at 0.4 mm for open systems and 0.4 mm for closed systems. Tablet print speed including infill print speed and outer perimeter print speed were all maintained at 30 mm/s. 

### 2.4. Characterization of Filaments and Tablets

#### 2.4.1. Fourier Transform Infrared Spectroscopy (FTIR)

The FTIR spectra of pure pregabalin, HPMCAS-HG, PEG-400, physical mixture, filament and tablet were obtained using Nicolet iS10 (Thermo scientific, Waltham, MA, USA). The scan’s frequency range was recorded as 400–4000 cm^−1^. 

#### 2.4.2. X-ray Powder Diffractometry (XRPD)

The crystallinity of pure pregabalin and formulated filaments and tablets was characterized by X-ray diffraction using X-ray diffractometer D/Max-2500 (Rigaku, Japan) operating at 40 kV and 200 mA. The samples were analyzed from 2θ = 3 to 45° at a step of 0.02° and a scan speed of 0.5°/min.

#### 2.4.3. Differential Scanning Calorimetry (DSC)

Pure pregabalin, physical mixture, filament and tablet were analyzed using DSC 4000 (Perkin–Elmer, Waltham, MA, USA) apparatus to study the effect of temperature. Samples were heated from 25 to 250 °C with a heating rate of 10 °C /min. Nitrogen gas was used as a purge gas with a flow rate of 20 mL/min. The degree of crystallinity (DOC) was calculated using the following Equation (1) [32]:

(1) $$DOC\left(\%\right)=\frac{\Delta Hs}{\Delta Hp\times W}\times 100$$

where *ΔHs* and *ΔHp* are the melting enthalpy of the test samples and pure pregabalin respectively. *W* is the mass fraction of pregabalin in the formulation.

#### 2.4.4. Thermogravimetric Analysis (TGA)

The thermal decomposition of API and formulations was carried by thermogravimetric analysis using a TA Q500 Auto-thermogravimetric analyzer (TA instruments, New Castle, DE, USA). Samples were heated from 25 to 250 °C with heating rate of 10 °C /min. Nitrogen gas was used as a purge gas with a flow rate of 40 mL/min. 

#### 2.4.5. Scanning Electronic Microscopy (SEM)

The surface morphology of filaments and tablets was studied by taking photographs using S-4800 SEM (Hitachi, Japan). 

#### 2.4.6. In Vitro Floating and Release Study

In vitro floating and in vitro release studies were conduction using the USP apparatus II paddle. Release study was carried out in accordance with the dissolution test of Korean pharmacopoeia. An amount of 500 mL of 0.06 N HCl buffer was used as dissolution media and temperature was set at 37 ± 0.5 °C with a rotational speed of 50 rpm. Samples were collected at predetermined times of 1, 2, 4, 6, 8, 12, and 24 h. Collected samples were filtered using 0.45 µm syringe filter and analysis was done using HPLC at 210 nm. The composition of the mobile phase was 0.04 M ammonium phosphate ((NH_4_)_2_HPO_4_) buffer solution: acetonitrile: methanol = 84:5:11 containing 5 mM sodium 1-octanesulfonate, and a flow rate was adjusted (0.7 min/mL) so that the retention time of pregabalin was about 6.66 min. A column was a stainless-steel column with an internal diameter of about 4.6 mm and a length of about 250 mm, packed with 5 µm-octadecylsilyl silica gel for liquid chromatography. The release study results were fitted to various kinetic models such as Zero-order [33], First-order [34,35], Higuchi [36], and Hixon-Crowell [37]. Finally, the statistical analysis for comparison of the release profiles of optimized formulation (F6) and marketed formulation Lyrica^®^ CR was done using a model independent approach: fit factor [38]. Moore and Flanner developed two equations to calculate the similarity and differences in the percentage (%) of drug dissolved per unit time between two dissolution profiles [39]. The similarity factor (*f_2_*) gives the similarity in the percent (%) dissolution between the two curves, which is calculated as a logarithmic reciprocal of the square root transformation of the sum of the squared error as shown in Equation 2.

(2) $$f_{2}=50\times \log{}^{\circ}\{{\left[1+(\frac{1}{n})\sum_{t=1}^{n}{\left(R_{t}-T_{t}\right)}^{2}\right]}^{-0.5}\times 100\}$$

where *R_t_* and *T_t_* are the cumulative percentage of drug dissolved at each of the selected ^‘^*n*^’^ time points of the commercial and optimized formulation, respectively. In this study, we used similarity factor (*f*_2_) to compare the dissolution profiles of optimized formulation and commercial product. The dissolution profiles are considered similar when *f*_2_ is between 50 and 100.

## 3. Results and Discussion

3D printing with FDM technology requires filaments as the starting material, which have the desired composition of raw materials required to print the final object. Various commercial filament makers are available for this process. However, in our study, HME technology was used to prepare pregabalin-loaded filaments. HME technology has widely been used in pharmaceutics to prepare solid dispersions of drugs with poor solubility [40]. Nevertheless, HME has also been extensively used and studied to prepare various pharmaceutical grade filaments [17,41]. Different batches of filaments were prepared with various compositions, as mentioned in Table 1, and filaments were accepted based on the feasibility of extrusion during the HME process and printing, as mentioned in Table 2.

Filament preparation and printing involves various process parameters that determine the result of the object to be printed. One of the variables that had a significant effect on the final printed object was the uniformity of the filament diameter. Commercially prepared filaments are available in 1.75 mm and 3 mm of diameters with deviations of ± 0.05 mm. However, prepared filaments were found to have a diameter of 1.5 mm, which is thinner than the commercially available and recommended filament size. Nevertheless, a slight change of print setting with filament diameter and flow rate of the filament from 100% to 120% made it possible to print out the tablets [42]. However, it is still important to have the filaments of a uniform diameter, as this will result in serious complications during extrusion. Under- or over-extrusion of the materials due to an inconsistent diameter of filaments is one of the major problems, along with difficulties associated with gripping of the filaments in the extruder, which causes coiling and breaking of the filament. Therefore, it is suggested to keep filament tolerance under ± 0.05 mm. For uniformity of the optimized filaments, we checked the thickness every 5-cm distance and the deviation was found to be ± 0.023. Another important parameter is the stiffness of the filaments. Filaments should have enough mechanical strength to not break in the feeding gear [41]. However, extremely flexible filaments also possess complications similar to an inconsistent diameter, such as under- or over-extrusion and coiling, along with stringing or oozing of extruded materials and weak infill. 

**Figure 2:**
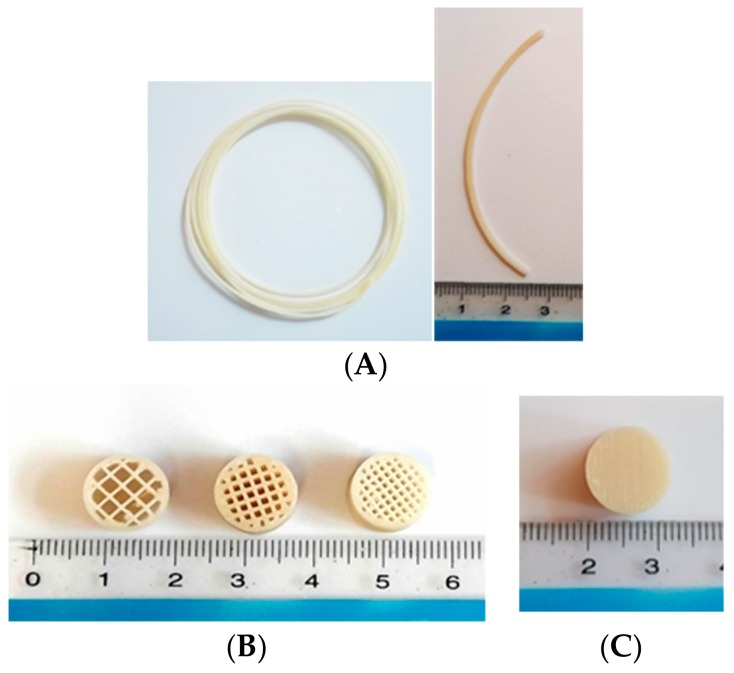
Prepared filaments and tablets. Drug loaded filaments (**A**), printed open system tablets with 25%, 50% and 75% infill left to right (**B**), and printed closed system tablet (**C**).

In our case, these problems were solved by using direct a drive extruder instead of a bowden extruder, using low print speed and heated bed and also controlling the feed tension, extrusion temperature and retraction. Finally, the filament prepared with 40% HPMCAS, 50% API and 10% PEG 400 (FIL-8) was used as optimized filament. Tablets were printed according to the design shown in Figure 2. Tablets were found to have uniform weight with a deviation within the range (Table 3). The uniformity of the diameter of the filament affected the weight variation as well. Tablets prepared with uniform filaments resulted in uniform weight tablets. Printed tablets showed very high mechanical strength and were impossible to test using a conventional hardness tester and the friability was completely zero, which is common in case of various FDM printed formulations [37,38].

**Figure 3:**
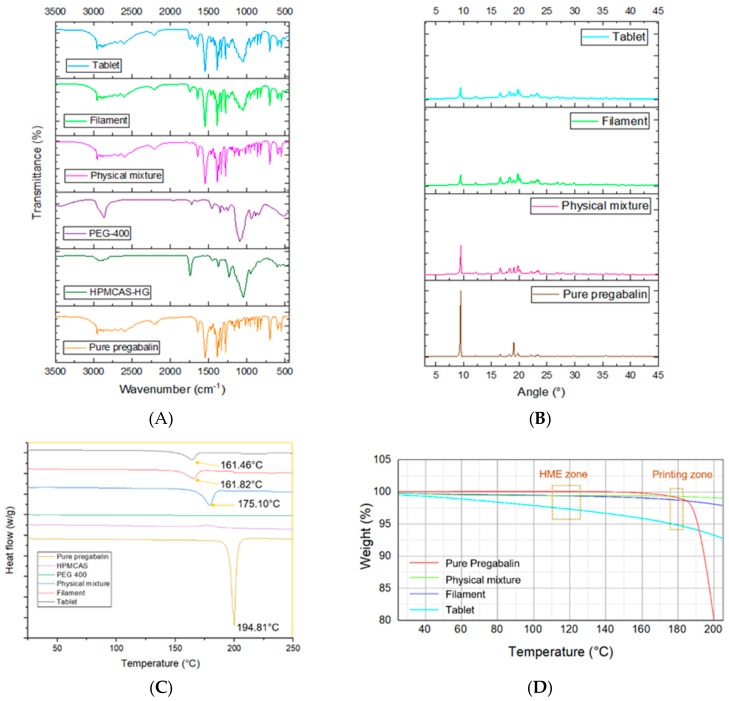
Physical characterization of printed tablets. Fourier-transform infrared spectroscopy (**A**), X-ray powder diffractometry (**B**), differential scanning calorimetry (**C**), and thermogravimetric analysis (**D**).

The FT-IR Spectrum of pure pregabalin and its physical mixture with polymers and different excipients are shown in Figure 3A. Pure pregabalin showed peaks at 2954.13 cm^−1^ (C–H stretch), 1642.69 cm^−1^ (N–H bend, NH_2_ scissoring), 1544.23 cm^−1^ (N–O asymmetric stretch), 1469.62 cm^−1^ (C–H bend), 1333.18 cm^−1^ (N–O symmetric stretch), 1277.44 cm^−1^ (C–O stretch), and 932.16 cm^−1^ (O–H bend) which was similar to peaks found in previous study [43]. The physical mixture, prepared filament and tablets had almost superimposed peaks, except for few small changes in peaks 1642 cm^−1^ to 1643 cm^−1^, 1544 cm^−1^ to 1546 cm^−1^ in the case of filament and 1545 cm^−1^ in the case of tablets, 1277 cm^−1^ to 1278 cm^−1^, and 1469 cm^−1^ to 1468 cm^−1^ in case of tablets. This shows that there was no significant interaction between API and the polymers. To further study the interaction between polymers with API and the effect of mechanical and thermal processes on drug crystallinity, XRPD and DSC were used. 

The XRPD pattern of pure pregabalin at 2 θ shows characteristic peaks at 4.7, 9.4, 18.20, 19.04, 19.75, 22.15, and 35.58 (Figure 3B). The characteristic peaks were also seen in the physical mixture, which was reduced in filaments and tablets. XRPD data reveals that pregabalin remained at least partly crystalline upon extrusion and printing which is in consistent with the results of DSC. The DSC graph shows the endothermic peak of pure pregabalin at 194.81 °C (Figure 3C). The endothermic had a negative shift to 175.10 °C in the physical mixture which further shifted to 161.82 °C and 161.46 °C. The degree of crystallinity was found to be 90%, 50% and 39% for the physical mixture, extruded filament and printed tablets, respectively. The extrusion process and printing seem to have had less effect on the crystallinity of pregabalin as the operating temperatures were lower than the melting point of API. FDM technology involves high temperatures in both the filaments-making process and the printing process. Although these processes require short exposure to high temperature, significant thermal degradation can be found in the case of thermolabile drugs and polymers [16,44]. The thermogravimetric analysis of API, physical mixture, filament and tablets were carried out as shown in Figure 3D. No significant mass loss was found from 115 to 125 °C (HME zone) and 180 °C (printing zone) for pure API, physical mixtures and filament. However, in the case of printed tablets, 2%–3% mass loss was found in the HME zone, while approximately 5% of mass loss was found in the printing zone. This could have been due to the repetitive exposure of printed tablets to high temperature, which resulted in the decomposition of polymers, causing weight loss. 

**Figure 4:**
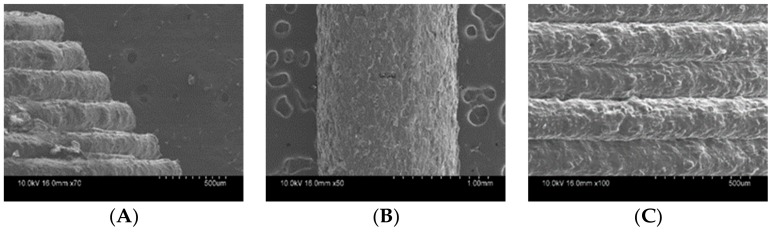
SEM image of filament and tablets. Outer surface of filament (**A**), side view of top layer (**B**), and center view of different layers (**C**).

SEM imaging shows the surface morphology of filaments and tablets (Figure 4). Filaments showed an irregular surface due to the low extrusion temperature, which resulted in incomplete melting of the drug and remained in crystalline form [45]. Drug loaded filaments prepared by HME are relatively rougher compared to commercial filaments [20]. In the case of the printed tablets, we can see prominent printed layers and uniform layer height. Uniformity in layer height determines the overall uniformity of tablet. However, the roughness/smoothness of a tablet does not have any effect on the tablet floating and release properties.

**Figure 5:**
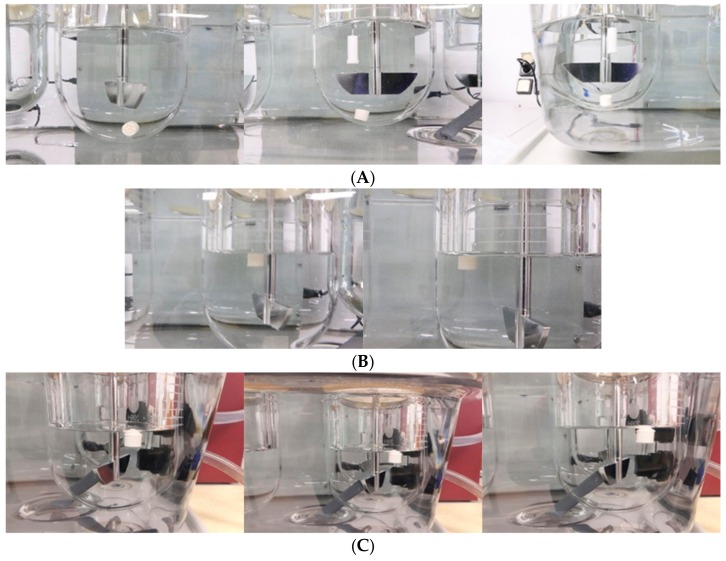
Floating study of prepared formulations. Open system (**A**), closed system (**B**), and optimized formulation (**C**) over 1, 8 and 24 h (left to right).

**Figure 6:**
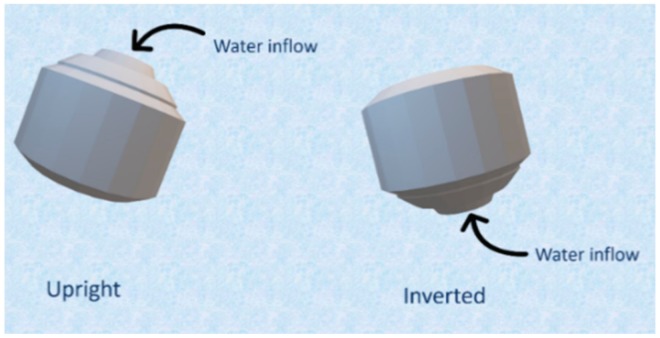
Floating mechanism of optimized formulation. The internal structure of tablet is composed of a grid infill with void space filled with air so that the tablet has low density, which helps in buoyancy of the tablet in media.

In vitro floating study of open and closed systems with different infill ratios were carried out (Figure 5). In a previous study, floating capacity and duration were found to be dependent on the density of the tablets [31], whereas, in our study, the floating study revealed a very high correlation between the presence of top/bottom layer and the floating capacity, with a very minimum dependency on the density. All the open systems (F1–F3) failed to show floating properties and sank immediately to the bottom of the vessel (Figure 5A), whereas the closed systems (F4 and F5) showed excellent floating properties and remained floating for >24 h (Figure 5B). Open systems have void spaces where water enters and replaces the air inside and increases the density of the tablets, causing it to sink in the media (Figure 6). On the contrary, in the case of closed system, water penetrates at a very slow rate so that the void spaces inside the tablet help to retain the buoyancy of the printed tablets, which remained true for the optimized formulation (Figure 5C) with closed system and open space on one side of the tablet. 

**Figure 7:**
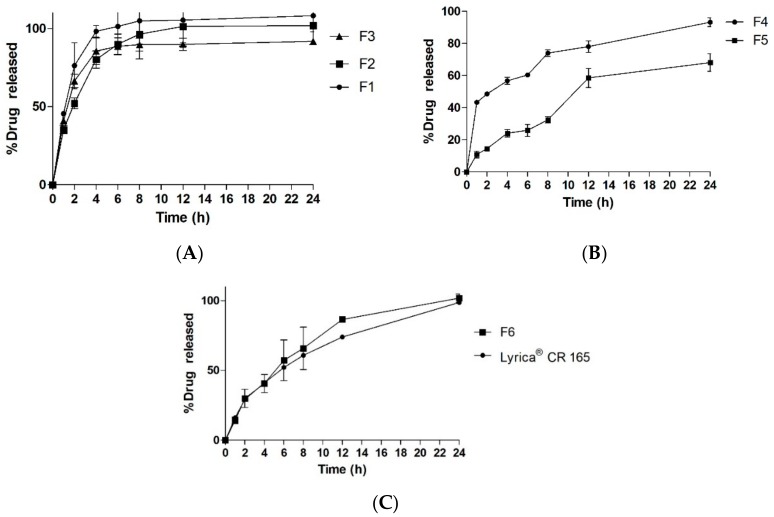
In vitro drug release study. Closed systems (**A**), open systems (**B**), and optimized formulation with commercial product (**C**).

**Figure 8:**
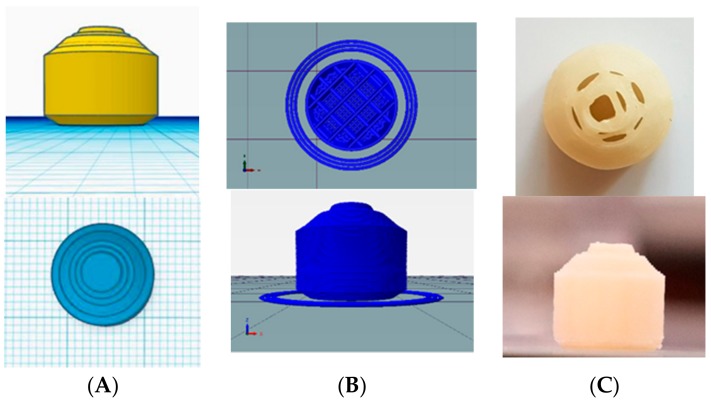
Top view and side view of optimized formulation. Design of unique shaped optimized formulation (**A**), slicing of tablets with 25% infill (**B**) and printed tablets (**C**).

The release study of closed formulations F4–F5 showed a relationship between the internal structure of the printed tablet and the drug release rate (Figure 7A). 3D printing has unique characteristics whereby the infill percentage and the infill pattern can be changed [46]. Infill is the internal structure of the printed object which determines the mechanical strength of object in the general 3D printing process. However, in the case of pharmaceutical formulations, the infill percentage of the formulation has been shown to play a major role in the kinetics of drug release in a number of studies. Drug release was significantly higher in formulations with low infill percentages [18]. In this study, tablets with low infill percentages showed faster release compared to tablets with high infill percentage. However, there was incomplete drug release in the case of tablets with a higher infill ratio. However, in the case of open system formulations F1–F3, the infill ratio did not have significant effect on the drug release (Figure 7B). A similar trend was noted in a previous study as well [31]. In addition, drug release was more controlled in the case of closed systems compared to open systems in which peak drug release was obtained around 6–8 h. The compactness of the tablet and surface area interaction with the media played huge role in drug release. Open tablets with low infill have higher access of media compared to closed tablets and tablets with high infill. This facilitated a faster release of the drug from the system. Thus, the optimized formulation was designed to mimic the advantages of both open and close systems (Figure 8). One side of the tablet was closed, and the other side was partially opened, and the infill was maintained at 25%. Moreover, the geometry of the formulation has shown to control the drug release [47]. This unique design (F6) helped to achieve complete drug release (Figure 7C) while retaining its floating ability for 24 h. The closed bottom of the tablet helped the buoyancy of the tablet and the partially opened top layer allowed entry of water inside tablet in a controlled manner, which facilitated complete drug release over longer period which is in contrast to F1 although it had similar dimension and infill percentages. The use of polymer also plays role in controlling the release of drugs from a printed formulation [16,17]. In a previous study, formulation prepared with HPMCAS HG was not completely dissolved even after 24 h and drug release was pH-sensitive as the enteric polymer was distributed into the matrix rather than as a coating layer in conventional formulations [45]. This phenomenon contributed to the extended release in the case of our formulation as well.

**Figure 9:**
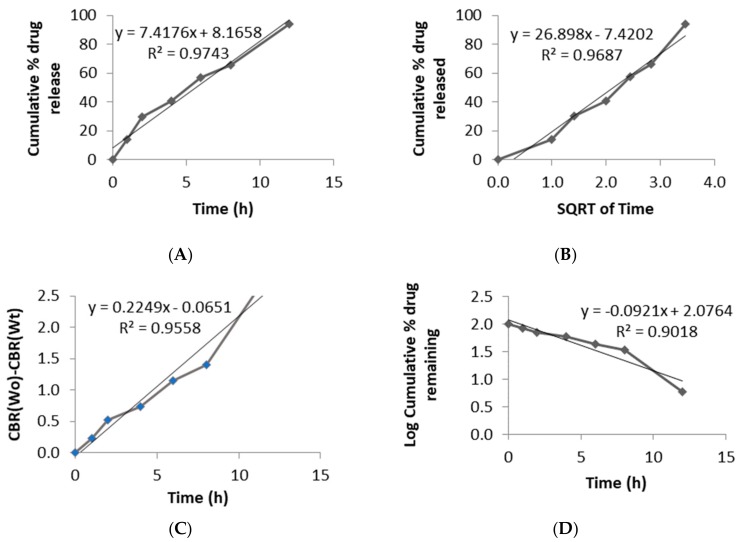
Drug release kinetics fitted to various models: Zero-order (**A**), Higuchi model (**B**), Hixon-Crowell model (**C**), and First-order (**D**).

To understand the release properties better, drug release data were fitted to different mathematical kinetic models (Figure 9). A single model could not define the drug release pattern from tablets and rather seemed to have a combination of different mechanisms. The regression value was found to be higher for the Zero-order and Higuchi models, suggesting that the release and diffusion rates were constant. Drug release for HPMCAS polymers have been found to be regulated by the drug diffusion and erosion polymer from the surface of the system [48]. Printed tablets did not show any changes in morphology and maintained their integrity during whole dissolution process.

Formulation F6 was compared to Lyrica^®^ CR 165 mg, a marketed product of Pfizer, and release profiles were compared using similarity factor (*f_2_*). The values of similarity factor (*f*_2_) for the batch F6 showed a maximum of 65.32 (Table 4); hence, it was selected as optimum batch. 

## 4. Conclusions

A uniquely shaped tablet was designed to formulate a floating gastro-retentive controlled release dosage form using FDM technology. This study proves the possibility of employing 3D printing technology to prepare a floating controlled release system of pregabalin. The feasibility of designing and printing tablets to meet a specific criterion using 3D printing technology has already been proven effective via various studies. Further development on design of formulations and resolution of printers to the extent where on-demand manufacturing and personalized medicine are possible will definitely change the future of pharmacotherapy.

## Figures and Tables

**Table 1:** Composition of filaments prepared.

Filaments	Pregabalin (%)	HPMCAS HG (%)	PEG 400 (%)	PVA (%)	HPMC E4 (%)	HPMC HME 15 LV (%)
FIL-1	-	-	-	-	100	-
FIL-2	-	-	-	10	90	-
FIL-3	-	-	10	-	90	-
FIL-4	25	-	10	-	-	65
FIL-5	25		5	-	-	70
FIL-6	50	20	10	-	-	20
FIL-7	25	65	10	-	-	-
FIL-8	50	40	10	-	-	-

**Table 2:** Properties of filaments obtained from the hot melt extruder.

Filaments	Result	Remarks
FIL-1	Difficulty in extrusion	High viscosity of polymer clogged extruder nozzle
FIL-2	Difficulty in printing	Filaments clogged print head due to gluey consistency after melting
FIL-3	Difficulty in printing	Filaments hardened with elevated temperature while printing
FIL-4	Difficulty in printing	Very flexible filaments
FIL-5	Difficulty in extrusion	Low concentration of plasticizer
FIL-6	Difficulty in printing	Very flexible filaments
FIL-7	Suitable for extrusion and printing	Filaments of enough strength and flow
FIL-8	Suitable for extrusion and printing	Filaments of enough strength and flow

**Table 3:** Characteristics and evaluation of printed tablets.

Formulation	Dimension (mm)	Infill (%)	Shell Thickness (mm)	Weight (mg)	Density (g/cm^3^)	Drug Content (mg)	Drug Loading (%)
F1	12 × 8	25	0.6	361.45 ± 0.35	0.40 ± 0.0003	168.22 ± 3.71	93.08 ± 1.54
F2	12 × 8	50	0.6	470.50 ± 8.48	0.52 ± 0.0091	227.75 ± 3.53	96.80 ± 1.26
F3	12 × 8	75	0.6	668.50 ± 27.57	0.74 ± 0.0304	322.75 ± 10.25	96.54 ± 3.22
F4	12 × 8	25	0.4	498.60 ± 4.52	0.55 ± 0.0050	235.95 ± 7.70	94.64 ± 3.03
F5	12 × 8	50	0.4	691.00 ± 14.14	0.76 ± 0.0156	335.47 ± 7.10	97.08 ± 2.06
F6	12 × 8	25	0.4	475.00 ± 2.57	-	234.90 ± 12.97	98.52 ± 5.40

**Table 4:** Comparison of prepared formulations with commercialized products using similarity factor (*f_2_*).

Formulations	F1	F2	F3	F4	F5	F6
**Lyrica^®^ CR 165**	19.53	26.9	25.7	40.88	37.2	65.32
